# Artemether-Lumefantrine versus Dihydroartemisinin-Piperaquine for Treatment of Uncomplicated *Plasmodium falciparum* Malaria in Children Aged Less than 15 Years in Guinea-Bissau – An Open-Label Non-Inferiority Randomised Clinical Trial

**DOI:** 10.1371/journal.pone.0161495

**Published:** 2016-09-20

**Authors:** Johan Ursing, Lars Rombo, Amabelia Rodrigues, Poul-Erik Kofoed

**Affiliations:** 1 Projecto de Saúde de Bandim, Indepth Network, Bissau, Guinea-Bissau; 2 Department of Microbiology, Tumor and Cell Biology, Karolinska Institutet Nobels väg 16, 171 65, Stockholm, Sweden; 3 Department of Infectious Diseases, Danderyds Hospital, Stockholm, Sweden; 4 Centre for Clinical Research, Sörmland county council, Eskilstuna, Sweden and Uppsala University, Uppsala, Sweden; 5 Department of Paediatrics, Kolding Hospital, Kolding, Denmark; Centers for Disease Control and Prevention, UNITED STATES

## Abstract

**Background:**

Artemether-lumefantrine (AL) was introduced for treatment of uncomplicated malaria in Guinea-Bissau in 2008. Malaria then resurged and recurrent malaria after treatment with AL and stock-outs of AL were common. This study therefore aimed to assess the efficacy of AL and identify an alternative second line antimalarial. Dihydroartemisinin-piperaquine (DP) was chosen as it has been shown to be safe and efficacious and to reduce the incidence of recurrent malaria.

**Methods and Findings:**

In a multicentre randomised open-label non-inferiority clinical trial, AL or DP were given over 3 days to children aged 6 months-15 years with uncomplicated *P*. *falciparum* mono-infection. Intake was observed and AL was given with milk. Children were seen on days 0, 1, 2 and 3 and then weekly days 7–42. Recurring *P*. *falciparum* were classified as recrudescence or new infections by genotyping. Between November 2012 and July 2015, 312 children were randomised to AL (n = 155) or DP (n = 157). The day 42 PCR adjusted per protocol adequate clinical and parasitological responses were 95% and 100% in the AL and DP groups respectively, Mantel-Haenszel weighted odds ratio (OR) 0.22 (95% CI 0–0.68), p = 0.022. In a modified intention to treat analysis in which treatment failures day 0 and reinfections were also considered as treatment failures adequate clinical and parasitological responses were 94% and 97% (OR 0.42 [95% CI, 0.13–1.38], p = 0.15). Parasite clearance and symptom resolution were similar with both treatments.

**Conclusions:**

Both treatments achieved the WHO recommended efficacy for antimalarials about to be adopted as policy. DP was not inferior to AL for treatment of uncomplicated *P*. *falciparum* malaria in Guinea-Bissau.

**Trial Registration:**

ClinicalTrials.gov NTC01704508

## Introduction

*Plasmodium falciparum* causes malaria and approximately 580 000 deaths each year [1]. Due to widespread resistance to antimalarial monotherapies, the World Health Organization recommends artemisinin-based combination therapy (ACT) for treatment of uncomplicated *P*. *falciparum* malaria. Artemether-lumefantrine (AL), dihydroartemisnin-piperaquine (DP) and artesunate-amodiaquine are the ACTs of primary relevance for use in Africa [2].

When AL was introduced for treatment of uncomplicated malaria in Guinea-Bissau in 2008, its efficacy was 97% [3]. Despite this, malaria resurged between 2008 and 2012 [4]. Data suggests that this increase was due to poorer than expected effectiveness of AL (unpublished data), unavailability of AL due to stock-outs and to increased use of the second-line drug quinine [4]. Quinine is typically given for 3 instead of 7 days in Guinea-Bissau and has very poor efficacy when used in that way [5]. Guinea-Bissau needs an alternative ACT for use when AL is not available or fails. Furthermore, concurrent use of multiple first line therapies may delay the development of drug resistance [6].

DP is taken once daily and has been shown to be safe, well tolerated and highly efficacious (>95%) in several African settings [7–10]. DP also protects from re-infection for longer time than AL and has thereby been linked to reduced incidence of uncomplicated and complicated malaria infections as well as hospitalizations in high transmission settings [8, 11]. DP has however, never been used in Guinea-Bissau. Artesunate-amodiaquine is also a potential ACT for use in Guinea-Bissau. However, amodiaquine tolerant *P*. *falciparum* exist in the country and the incidence has increased in recent years [12, 13]. We therefore conducted this non-inferiority randomised clinical trial to determine the efficacy and safety of AL and DP for the treatment of uncomplicated *P*. *falciparum* malaria in Guinea-Bissau.

## Methods

### Ethics and registration

The study was conducted in accordance with the Declaration of Helsinki and Good Clinical Practice. Written informed consent was obtained from all parents/legal guardians. The Ministério da Saúde Pública in Guinea-Bissau (Ref (2)/CNES/INASA/2012 and ref 011/CNES/INASA/2013) gave ethical approval. The regional ethics committee in Stockholm, Sweden approved the molecular analyses (2011/832-32/2). The study was registered at ClinicalTrials.gov (NCT01704508).

### Study site and period

The Bandim Health and Demographic Surveillance Site (HDSS) was established in 1978 and covers a 16 km^2^ area of suburban Bissau, the capital of Guinea-Bissau [14]. Three health centres and the national hospital approximately 4 km away serve the population. The study was conducted at The Bandim and Belem Health Centres. Each centre has a small laboratory manned by trained laboratory technicians equipped with microscopes and rapid diagnostic tests for malaria. Nurses routinely see paediatric patients in the mornings and as emergencies during afternoons and weekends.

*P*. *falciparum* was mesoendemic during the 1990’s and then gradually decreased reaching the lowest recorded incidence in 2007. Malaria resurged between 2008 and 2012. Following mass distribution of insecticide treated bed-nets in November 2011 the malaria incidence decreased again [4]. Malaria transmission is now very seasonal with a peak in November and minimal transmission February-July. This study was conducted between November 2012 and July 2015 covering 2.5 transmission seasons.

### Patients

Inclusion criteria were: Mono-infection with *P*. *falciparum* without signs of severe malaria or danger signs. Parasite density of 800–200 000 trophozoites/μL, axillary temperature >37.5°C or a history of fever during the previous 24 hours, age 6 months to 15 years, haemoglobin >50g/L, no other significant illness, no reported intake of antimalarials during the past week and residence within the study area.

### Study design

This was a multicentre, randomised open-label non-inferiority clinical trial comparing DP with AL for the treatment of children with uncomplicated malaria. Randomisation was achieved by mothers drawing a sealed card with either Eurartesim or Coartem written on it from an envelope containing 10 of each card. Study nurses allocated treatment according to the drawn card. Cards and envelopes were prepared by the investigators.

Medication was given and supervised by an experienced nurse. Tablets were crushed for young children unable to swallow them whole. Mothers were encouraged to continue nursing. AL was given with approximately 200 mL of milk.

AL containing 20 mg of artemether and 120 mg of lumefantrine (Ipca Laboratories^TM,^ Mumbai, India) were purchased from the national drug dispensary (CECOM). 1–4 tablets per dose were prescribed according to body weight and given at approximately 0, 8, 24, 36, 48, and 60 hours after inclusion.

DP tablets containing 20/160 mg or 40/320 mg dihydroartemisinin/piperaquine (Eurartesim^®^) were provided free of charge by Sigma-Tau, Rome, Italy. DP was dosed according to body weight once daily on days 0, 1 and 2. The daily number of tablets of Eurartesim^®^ 20/160 mg were 0.5 (5 to <7kg) or 1 (7 to <13kg). The daily number of tablets of Eurartesim 40/320 mg were 1 (13 to <24kg), 2 (24 to <36) or 3 (36 to <75kg).

Doses were repeated to children vomiting within 30 min of treatment. If a child vomited again during the next 30 min, the child was given quinine intramuscularly and withdrawn. Concomitantly prescribed drugs were administered and recorded. *P*. *falciparum* detected after day 7 were treated with AL.

Children were seen twice daily on days 0, 1 and 2 and then once on days 3, 7, 14, 21, 28, 35 and 42. Clinical condition was assessed, temperature measured and mothers were questioned about their child’s general condition and the occurrence of fever, any symptoms, hospital admission or intake of any drugs each time a child was seen. Daily on days 0, 1, 2, 3 and 7, patients and / or their care-giver were asked if the patient had any of the following symptoms during the past 24 hours: Fever, convulsions, joint pains, diarrhoea, stomach pain, itch, rash, palpitations, inability to eat, sleep and drink, their general condition and any other symptoms. Symptoms were graded as not present, moderate or severe. In addition, the attending nurse assessed patient’s general condition as well, ill or very ill and measured the temperature. If a child was not seen during a weekly visit, a health worker phoned the parent/guardian or revisited the house the following two days. Children were given free medication and were encouraged to return at any time if their health deteriorated. If a child was admitted to hospital, the diagnosis and treatment was recorded. Each time a child was seen, including unscheduled visits to the health centres, Giemsa stained thick and thin smears were made to identify and quantify any malaria parasites. Blood was put on to filter-paper (Whatman 3MM) for later genotyping whenever a slide was made except for days 1 and 2 and when no parasites were found during unscheduled visits. Filter-papers were put into separate sealed plastic bags to avoid contamination and stored with a desiccant (Minipax^®^, Sigma-Aldrich^®^).

### Laboratory methods

Children with symptoms suggestive of malaria were screened using rapid diagnostic tests (First Response^®^) and Giemsa stained thick and thin smears using finger prick blood. Species were identified and asexual parasite density quantified (per 200 white blood cells) using 1000 times magnification and a sunlit microscope. A slide was considered negative after examination of 100 high power fields.

Haemoglobin was measured using a HaemoCue (Ängelholm, Sweden) on days 0, 3 and 42. Moderate anaemia was defined as haemoglobin <100 g/l in children under 5 years of age and <110 g/l in older children. For severe anaemia the corresponding haemoglobin values were 70 and 80 g/l, respectively.

DNA was extracted from two 3 mm punches of blood soaked filter-paper using a cheelex^TM^ based extraction method. DNA was frozen in aliquots at –20°C until amplification by PCR. New and recrudescent infections were distinguished using a nested PCR protocol according to WHO recommendations [15]. Merozoite surface proteins 1 and 2 and *P falciparum* glutamate rich protein were analysed in matching day 0 samples and day of recurring *P*. *falciparum*. PCR products were resolved on agarose gels (Amresco, Solon, OH), stained with ethidium bromide and visualized under UV transillumination (GelDoc^®^, Biorad, Hercules, Ca, USA).

### Treatment outcomes

We used the WHO criteria to identify early treatment failures (ETF), late clinical failures (LCF), late parasitological failures (LPF) and adequate clinical and parasitological response (ACPR) [16]. Briefly, ETF was defined as the development of danger signs or severe malaria on days 1–3 in the presence of parasitaemia. PCR verified recrudescence of *P*. *falciparum* between days 7–42 was defined as LCF if the subject had symptoms of malaria and LPF if there were no symptoms. In the per protocol analysis ETF and PCR corrected LCF and LPF were considered as treatment failures whereas reinfections (identified by PCR genotyping) were censored. In the intention to treat analysis day 0 treatment failures, ETF and all reparasitaemias were considered as treatment failures. Patients were censored due to loss to follow up if they had moved from the study area or were not seen on two consecutive weekly visits. Patients were censored due to withdrawal of consent if they refused blood sampling for malaria diagnosis at any time during the study period.

The primary endpoint was PCR adjusted per protocol ACPR day 42. Secondary endpoints included intention to treat ACPR day 42, haemoglobin recovery, fever and parasite clearance and drug tolerability.

### Statistics

Assuming a PCR corrected per protocol day 42 ACPR of 97% (based on data from a trial with AL in 2008), 5% significance level (alpha), 80% power (1-beta), a non-inferiority limit (d) of 5% the sample size was calculated to be 288 not accounting for loss to follow up. Sample sizes were calculated to be 317 and 346 assuming a 10% and 20% loss to follow up, respectively.

Data analysis was done using Stata 12.0 and Epi Info™. We planned to compare treatment arms using a Cox proportional hazards model but this was not possible as the efficacy of DP was 100% in the per protocol analyses. Treatment outcomes were therefore compared using Mantel-Haenszel weighted odds ratio (OR). Nevertheless, the effect of sex, age group (<5, 5–9 and 10–14 years), parasite density (<50000, 50000–99999 and >99999 *P*. *falciparum*/μL) and study site were assessed in the intention to treat analysis using a Cox proportional hazards model. No predictor had a significant effect. Data from the Mantel-Haenszel analysis only are therefore presented. Quantile regressions with bootstrapping (100 repetitions) were used to compare parasite densities, body temperatures and haemoglobin values. Quantile regressions were used as parasite density was not normally distributed and because we wished to analyse medians to minimise the effect of outliers. Symptoms, the proportion of children with anaemia, the proportion of children with parasitaemia and gametocytes on specific days were analysed using Fisher’s exact test.

## Results

**Fig 1:**
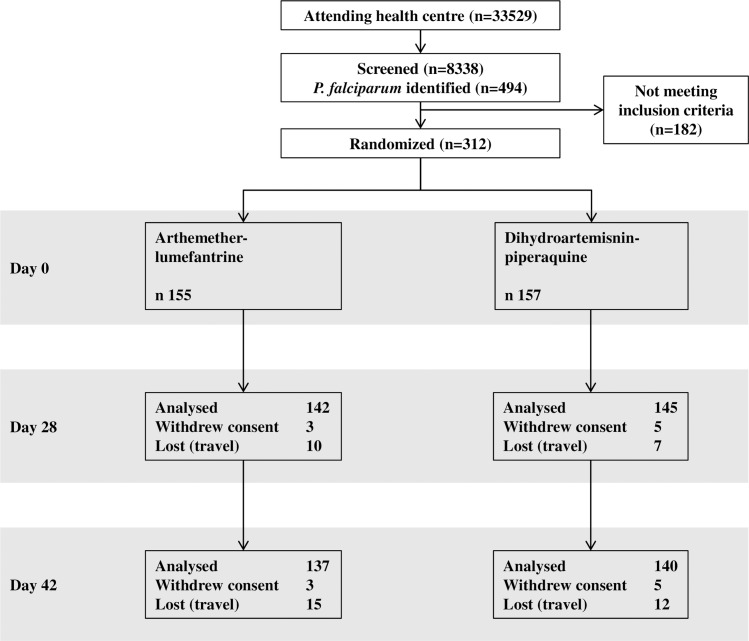
Patient Flow. All withdrawals of consent were due to patients not wanting more blood samples to be taken. All losses to follow up were due to patients travelling. Patients that travelled or withdrew consent contributed time to the Mantel-Haenszel weighted odds ratio calculation until they were censored.

A total of 8338 children were screened for malaria of which 494 were found to have *P*. *falciparum* on examination of thick and thin smears. Inclusion criteria were not fulfilled by 182 children due to residence outside the study area (primarily), low parasite density or unwillingness to participate. The study was stopped when 312 children had been included because a new study was due to start. Participant flow is shown in Fig 1. All withdrawals of consent were due to parents/guardians not wanting more blood sampling to be done. In the AL and DP arms loss to follow up by day 42 was 10% (n = 15) and 8% (n = 12), respectively. Losses to follow up were due to children travelling. Baseline characteristics (Table 1) were similar between groups even when stratified into age groups except for gender. Gender was not associated with severity of disease, other symptoms or parasite density.

**Table 1:** Basic characteristics at inclusion.

Characteristic	Artemether-lumefantrine	Dihydroartemisinin-piperaquine
**Median age (years)**	10 (7–12)	10 (7–12)
**Weight (Kg)**	27 (20–34)	27 (20–35)
**Sex (male:female)**	91:64	63:94
***P*. *falciparum* / μL**	18800 (7600–47619)	23121 (11400–57971)
**Axillary temperature °C**	38.1 (37–39.1)	38.3 (37–39.2)
**Haemoglobin (g/dL)**	112 (100–123)	113 (102–122)
**Moderate or severe anaemia**	37%	37%
**Assessed clinical condition**		
**Very ill**	29 (19%)	32 (21%)
**Moderately ill**	124 (80%)	120 (78%)
**Not ill**	1 (1%)	2 (1%)

Interquartile ranges are shown in brackets

Moderate anaemia was defined as haemoglobin <100 g/l in children under 5 years of age and <110 g/l in older children. For severe anaemia the corresponding haemoglobin values were 70 and 80 g/l, respectively.

The attending nurse assessed patient’s general condition as well, ill or very ill.

Treatment outcomes are shown in Table 2. The PCR adjusted per protocol day 42 ACPRs were 95% (95% confidence interval [CI], 91–98%) and 100% (CI could not be calculated) for AL and DP respectively. In numbers this was 7 versus 0 treatment failures and 128 versus 136 ACPRs in the AL and DP arms, respectively. DP was significantly less likely to result in treatment failure OR 0.22, (95% CI, 0–0.68, p = 0.022). The non-inferiority margin was 90% and this was most probably achieved but as 95% confidence intervals could not be calculated non-inferiority was in a strict sense not shown. In the modified intention to treat analysis in which treatment failures on day 0 and all recurrent infections were considered as treatment failures the ACPRs were 94% (95% CI, 89–97%) and 97% (95% CI, 93–99%) for AL and DP, respectively (OR 0.43, [95% CI, 0.10–1.56], p = 0.15). The non-inferiority margin was 89% in this analysis and as DPs lower 95% CI was 93% DP was non-inferior and not superior.

**Table 2:** Treatment outcome.

	Artemether-lumefantrine	Dihydroartemisinin-piperaquine	Weighted odds ratio
**Number**	155	157	
**ACPR**	128	136	
			
**Per protocol ACPR**	95%	100%	0.22 (95% CI 0–0.68)*, p = 0.022
**Early treatment failure**	4	0	
**Late clinical failure**	3	0	
			
**Modified intention to treat ACPR**	94%	97%	0.43 (95% CI, 0.10–1.56), p = 0.15
**Early treatment failure**	6	2	
**Late clinical failure**	3	0	
**New infection**	0	2	
			
**Withdrawal of consent**	3	5	
**Lost to follow up**	15	12	

ACPR Adequate clinical and parasitological response

*There were two treatment failures in both the DP and AL group on day 0 that were treated as wrong inclusions in the per protocol analysis.

In the modified intention to treat analysis treatment failures on day 0 and new infections were considered as treatment failures. Withdrawal of consent and loss to follow up were censored in the per protocol and intention to treat analyses.

There were two treatment failures (considered as ETF in the intention to treat analysis) on day 0 in both the AL and DP arms and four ETF on day one in the AL arm. All eight ETF were admitted to hospital (six to the national hospital, one to the Bandim health centre and one to a different hospital). The number of ETF were not significantly different between treatment arms (Fishers exact p = 0.17). No other children received antimalarial treatment outside of the study protocol.

There were three recrudescent infections in the AL arm occurring on day 14 (n = 2) and 21 and none in the DP arm. There were two new infections (day 35 and day 42) in the DP arm and none in the AL arm

Median haemoglobin values decreased significantly between day 0 and day 3 and increased significantly between day 3 and day 42 in both treatment arms (p<0.001) as shown in Table 3. The proportion of children with moderate (p<0.01) and severe (p<0.02) anaemia changed similarly. Median haemoglobin values and the proportion of children with moderate or severe anaemia did not differ significantly between treatment groups.

**Table 3:** Median haemoglobin values (g/L) and proportion of children with anaemia on days 0, 3 and 42.

		Artemether-lumefantrine	Dihydroartemisinin-piperaquine	All
**Day 0**	Median Hb (95% CI))	112 (107–117)	113 (110–1116)	113 (110–116)
	Moderate anaemia	36%	36%	36%
	Severe anemia	1%	1%	1%
**Day 3**	Median Hb (95% CI)	106 (102–110) p = 0.08	106 (104–109) p = 0.001	106 (104–108) p<0.001
	Moderate anaemia	53%	56%	55%
	Severe anaemia	10%	7%	8%
**Day 42**	Median Hb (95% CI)	117 (115–120) p<0.001	118 (115–121) p<0.001	118 (116–120) p<0.001
	Moderate anaemia	23%	23%	23%
	Severe anaemia	0%	1%	0.4%

Moderate anaemia was defined as haemoglobin <100 g/l in children under 5 years of age and <110 g/L in older children. For severe anaemia the corresponding haemoglobin values were 70 and 80 g/L, respectively.

Median haemoglobin values and 95% confidence intervals were calculated and compared using quantile regression. Proportions were compared using Fishers exact test.

Median haemoglobin decreased significantly between day 0 and day 3 and increased significantly between day 3 and day 42 in both treatment arms (p<0.001). The proportion of children with moderate (p<0.01) and severe (p<0.02) anaemia changed similarly. Median haemoglobin values and the proportion of children with moderate or severe anaemia did not differ significantly between treatment groups.

Median parasite densities were 18800 (95% CI, 8710–28890) and 23121 (95% CI, 15577–30666) *P*. *falciparum*/μl on day 0 in the AL and DP groups, respectively. These decreased to 1280 (95% CI, 846–1714) and 480 (95% CI 332–628) *P*. *falciparum*/μl on day 1 (p<0.001). The proportion of children with parasitaemia on days 0, 1, 2 and 3 and rate of fever clearance were not significantly different between treatment arms. Stratifying by age did not affect this.

Gametocyte carriage was less than 1% in both treatment arms throughout the study and did not vary significantly with treatment. No gametocytes were seen on days 35 and 42.

### Symptom resolution and adverse events

On days 0, 1, 2 and 3, the proportion of patients assessed by the study nurse as ill or very ill were 99%, 70%, 17% and 2% in the AL arm and 99%, 60%, 14% and 3% in the DP arm. On day 0, 1, 2 and 3 convulsions, joint pains, diarrhoea, stomach pain, itch, rash, palpitations, inability to eat, sleep and drink and/or any other symptoms during the past 24 hours were reported in 42%, 18%, 5% and 1% in the AL arm and 40%, 15% 3% and 1% in the DP arm. There were no significant differences. Except for recurrent parasitaemia, there were no severe adverse events during the follow up.

Treatment was repeated due to vomiting on day 0 in 4/155 and 7/157 children receiving AL and DP, respectively (p = 0.54). On days 1 and 2 treatment was repeated in one and zero children taking AL and one and two children taking DP, respectively.

## Discussion

This aim of this study was to assess and compare the efficacy of AL and DP in Bissau, Guinea-Bissau. The study was prompted by a perceived lack of effectiveness of AL when malaria resurged between 2008 and 2012 and a wish to identify a second line ACT [4]. The ≥95% PCR corrected efficacy of AL and DP allays fears of widespread AL resistance and shows that DP fulfilled WHO criteria for antimalarials to be introduced [2]. Furthermore, DP was shown to be non-inferior and not superior to AL in the non-inferiority analysis. Thus both AL and DP can be recommended for the treatment of uncomplicated malaria in Guinea-Bissau.

The per protocol day 42 PCR corrected efficacy of DP (100%) was significantly better than that of AL (95%). There were four ETFs and three LCFs in the AL arm versus none in the DP arm. Furthermore, the median parasite density was significantly higher in the AL arm (1280 Pf/μl) compared to the DP arm (480 Pf/μl) on day one. On the other hand, the majority of children cleared *P*. *falciparum* within 48 hours of starting treatment with AL or DP suggesting that *P*. *falciparum* remain sensitive to artemisinin derivatives. The numbers are small and data should be interpreted with caution, especially as we do not have data on drug concentrations. However, AL has previously been linked to an increased risk of persistent parasitaemia on day one compared to DP [17]. Furthermore, as shown in earlier studies, the frequency of genotypes associated with five-fold higher lumefantrine IC50 values was high (~75%) when AL was introduced in Guinea-Bissau [13, 18]. The results therefore highlight the importance of continued AL efficacy monitoring in Guinea-Bissau.

Lower incidence of uncomplicated and complicated malaria infections, fewer hospitalizations, better haemoglobin values and lower risk of developing gametocytaemia have been found in children treated with DP compared to AL [8, 9, 19]. These effects have been attributed to a post treatment prophylactic effect due to the longer terminal elimination half-life of piperaquine compared to lumefantrine. In our study DP was no better than AL at preventing reinfections and haemoglobin values did not differ between treatment groups. This was no doubt due to the currently low transmission intensity (there were only two reinfections) at our site in line with data from a meso-endemic area of Kenya [20]. When malaria resurged in Bissau (2008–2012), reinfections were common even during the short seasonal transmission period. A drug with a long post treatment prophylactic effect might limit onward transmission of malaria during epidemics. Based on previous data and the high efficacy of DP in this study it is possible that use of DP can minimise seasonal peaks of malaria in Guinea-Bissau.

Both drugs were well tolerated and fever clearance, symptom resolution and haemoglobin recovery were similar. DP has previously been linked to increased risk of vomiting in infants [21] and we found that the need to repeat treatment due to vomiting was non-significantly more common after the first dose of DP (n = 7) compared to AL (n = 4). However, vomiting was not seen with subsequent doses and no child was withdrawn due to vomiting indicating that both treatments were well tolerated.

As expected haemoglobin values were lower on day 3 than day 0. The day 3 haemoglobin value was also less likely to be effected by fever and dehydration as the clinical condition of the vast majority of children was assessed as good on day 3. Thus the increase of haemoglobin between days 3 and 42 is most probably a more accurate reflection of haemoglobin recovery than the day 0 and day 42 comparison commonly used.

### Conclusions

Both AL and DP fulfilled WHO criteria for treatment of uncomplicated *P*. *falciparum* malaria and can be recommended for use in Guinea-Bissau. DP was not inferior to AL for treatment of uncomplicated *P*. *falciparum* malaria. Haemoglobin recovery is better assessed by measuring haemoglobin on days 3 and 42 compared to days 0 and 42.

## Supporting Information

S1 File Consort 2010 Checklist. (DOC)

S2 File Improving anti-malarial treatment options in Guinea-Bissau. (DOC)
