# Supplementary material for: Artemether-Lumefantrine versus Dihydroartemisinin-Piperaquine for Treatment of Uncomplicated Plasmodium falciparum Malaria in Children Aged Less than 15 Years in Guinea-Bissau – An Open-Label Non-Inferiority Randomised Clinical Trial
Source: PLoS One. 2016 Sep 20;11(9):e0161495. doi: 10.1371/journal.pone.0161495 (PMC5030079; doi:10.1371/journal.pone.0161495)
Supplement: S2 File — (DOC) [file pone.0161495.s002.doc]

**Improving anti-malarial treatment options in Guinea-Bissau**

**Investigators**

From Guinea-Bissau: Amabelia Rodrigues^1^ MD, PhD a.rodrigues@bandim.org

From Denmark: Poul-Erik Kofoed^1,2^ MD, PhD. pekofoed@dadlnet.dk

From Sweden: Johan Ursing MD^1,3^, PhD. [johan.ursing@karolinska.se](mailto:johan.ursing@karolinska.se)

Lars Rombo^3,4^ MD, PhD. [lars.rombo@dll.se](mailto:lars.rombo@dll.se)

From Portugal: Isabel Veiga^5^ BSc. PhD. [maria.isabel.veiga@gmail.com](mailto:maria.isabel.veiga@gmail.com)

**Affiliations**

1. Projecto de Saùde de Bandim, Bissau, Guinea-Bissau
2. Kolding Sjukhus, Kolding, Danmark
3. Malaria Research Group, Retzius väg 10, Karolinska Institutet, Stockholm, Sweden
4. Clinical research centre, Sormland county council, Eskilstuna, Sweden
5. Domínio de Microbiologia e Infecção, ICVS, Universidade do Minho, Portugal

**Summary**

*Plasmodium falciparum* causes malaria and approximately 665 000 deaths each year. Chloroquine and sulphadoxine-pyrimethamine resistant *P. falciparum* are widespread. An artemisinin derivative combined with lumefantrine, amodiaquine or piperaquine is therefore recommended for the treatment of malaria in Africa. However, artemisinin resistance appears to be developing and resistance/tolerance to amodiaquine and lumefantrine exists.

We are presently monitoring malaria treatment in Guinea-Bissau. Preliminary data indicates that the effectiveness and availability of artemether-lumefantrine, the 1^st^ line drug, is poor and approximately 50% of children are treated with quinine. Consequently there is a need for another treatment option. Dihydroartemisinin-piperaquine has been shown to be efficacious and well tolerated in several African countries and is therefore such an option.

We propose to conduct clinical trials comparing the safety, efficacy and effectiveness of artemether-lumefantrine and dihydroartemisinin-piperaquine.

**Background**

*Plasmodium falciparum* causes malaria and approximately 665 000 deaths each year. Previously, chloroquine and sulphadoxine-pyrimethamine were the principle drugs for the treatment of malaria. Due to widespread resistance to these drugs[^1^](#_ENREF_1), the World Health Organization recommends that *P. falciparum* in Africa should be treated with artemisinin + amodiaquine, artemether + lumefantrine (AL) or dihydroartemisinin + piperaquine[^2^](#_ENREF_2). However, resistance to artemisinins appears to be developing, resistance to amodiaquine exists[^2^](#_ENREF_2) and treatment with AL rapidly selects for parasites with increased tolerance (5 times higher inhibitory concentration) to lumefantrine[^5-7^](#_ENREF_5).

In Guinea-Bissau, chloroquine remained efficacious until replaced by AL. This is explained by the use of a unique well tolerated high dose treatment schedule [^8-10^](#_ENREF_8). Since the introduction of AL in 2008, the number of children with malaria has increased manyfold in Bissau in contrast with other African countries. Our data suggests that this increase is due unavailability of AL, poorer than expected effectiveness of AL and to an increased use of the second-line drug quinine. Quinine is typically given for 3 instead of 7 days and has very poor efficacy when used thus[^11^](#_ENREF_11). To counter this, Guinea-Bissau needs an alternative cheap and easily dosed 2^nd^ line drug that can also be used when AL is not available or when funding for AL no longer exists.

Dihydroartemisinin+piperaquine (DP) is a safe and well tolerated artemisinin based combination[^12^](#_ENREF_12). DP is taken once daily and has been shown to be highly efficacious (>95%) in several African settings. DP also protects from re-infection for longer than AL[^14^](#_ENREF_14). As such this is a drug that could become an attractive treatment option in Guinea-Bissau. It has however, never been used in the country.

**Aim**

The overall aim is to determine the efficacy, effectiveness and safety of artemether + lumefantrine and dihydroartemisinin + piperaquine.

Specific aims are to

A) Conduct an efficacy study with artemether + lumefantrine and dihydroartemisinin + piperaquine

B) Conduct an effectiveness study with artemether + lumefantrine and dihydroartemisinin + piperaquine

**Method**

**A. Efficacy and safety of artemether + lumefantrine and dihydroartemisinin + piperaquine for the treatment of uncomplicated malaria in Guinea-Bissau**

**1. Objectives**

1. To measure the efficacy and safety of AL and DP in children aged 6 months to 12 years suffering from uncomplicated *P. falciparum* malaria.
2. To determine the capacity of each drug combination to protect against re-infection.
3. To differentiate recrudescence from re-infections using PCR based methods
4. To determine whole blood concentrations of lumefantrine and piperaquine the week before reparasitaemia
5. To determine haemoglobin values on days 0, 3 and 42
6. To determine differential white blood cell counts on days 0, 3, 7, 14 and 21
7. To determine genetic polymorphisms in *P. falciparum* causing reparasitaemia,
8. To culture parasites from 50 children for further characterisation of *P. falciparum* geno- and phenotypes.

**2. Methods**

**Study design**

This will be an open label, randomized, non inferiority trial conducted at the Bandim health centre, Guinea-Bissau. Children with uncomplicated malaria who meet study inclusion criteria will be enrolled, randomised to treatment with either AL or DP, treated on site and monitored for 42 days. The follow up will consist of a fixed schedule of check-up visits and corresponding clinical and laboratory examinations as in previous studies of ours. The proportion of children experiencing therapeutic failure during the follow-up period will be used to estimate the efficacy of the study drugs. PCR analysis will be used to distinguish between a true recrudescence due to treatment failure and episodes of re-infection.

**Study Site and population**

Children aged 6 months to 12 years with uncomplicated *P. falciparum* malaria that attend the Bandim health centre.

**Timing and duration of the study**

The study is expected to start in October 2012 and continue until 350 children are included which we believe will take approximately one year.

**Inclusion criteria**

A) Age ≥6 months, and <13 years. B) Mono-infection with *P. falciparum* detected by microscopy. C) Parasitemia of 1.000-200.000/µl asexual forms. D) Axillary temperature ≥37.5 ˚C or a history of fever within 24 hours. E) Ability to swallow oral medication.

F) Ability and willingness to comply with the study protocol for the duration of the study and to comply with the study visit schedule. G) Informed consent from a parent or guardian

**Exclusion criteria**

A) Signs or symptoms of severe malaria including the following symptoms

Prostration. Impaired consciousness. Respiratory distress. Repeated generalised convulsions (three or more per 24 hours or 2 witnessed seizures in 24 hours). Circulatory collapse. Abnormal bleeding. Jaundice. Haemoglobinuria (dark red/black urine). Severe anaemia (Haemoglobin <5g/dl). Hyperparasitaemia (>200.000/ µl asexual forms)

B) Presence of general danger signs in children under 5 including the following

Prostration. Respiratory distress. Haemoglobin <5g/dl. Two or more convulsions within 24 hours. Persistent vomiting

C) Presence of severe malnutrition. D) Any evidence of chronic disease or acute infection other than malaria. E) Regular medication which may interfere with antimalarial pharmacokinetics. F) History of hypersensitivity reactions or contraindications to AL, DP or quinine. G) Subjects with known cardiac arrhythmias or with congenital prolongation of the QTc-interval and patients who are taking medicinal products that are known to prolong the QTc interval as outlined in the summary of product characteristics for Eurartesim®. H) Domicile outside the study area.

**Loss to follow up**

If a child is not seen on the day of follow up the child will be sought at home on two consecutive days and if possible contacted by mobile phone. At each subsequent weekly follow up three new attempts to see the child will be made.

**3. Treatment**

**Antimalarial treatment**

Treatment with AL will be given according to a 6-dose regime shown below. Treatment with DP will be given according to a 3 dose regime. Tablets of AL (20 mg artemether and 120 mg lumefantrine) will be obtained from the manufacturer. Tablets of DP (Eurartesim®) with 160mg/20mg or 320mg/40mg if piperaquine/dihydroartemisinin will be obtained from Sigma-Tau**.**

Artemether-lumefantrine dosing

| **Body weight in kg**  **(age in years)** | **No. of tablets recommended**  **And approximate timing of dosing** | | | | | |
| --- | --- | --- | --- | --- | --- | --- |
|  | **0 h** | **8 h** | **24 h** | **36 h** | **48 h** | **60 h** |
| 5–14 (<3) | 1 | 1 | 1 | 1 | 1 | 1 |
| 15–24 (3–9) | 2 | 2 | 2 | 2 | 2 | 2 |
| 25–34 (9–14) | 3 | 3 | 3 | 3 | 3 | 3 |
| >34 (>14) | 4 | 4 | 4 | 4 | 4 | 4 |

Dihydroartemisinin-piperaquine dosing

| **Body weight in kg** | **Tablet strength PPQ/DHA**  **in mg** | **No. of tablets recommended**  **and approximate timing of dosing** | | | | | |
| --- | --- | --- | --- | --- | --- | --- | --- |
|  |  | **0 h** | **8 h** | **24 h** | **36 h** | **48 h** | **60 h** |
| 5 to <7 | 160/20 | 1/2 | 0 | 1/2 | 0 | 1/2 | 0 |
| 7 to <13 | 160/20 | 1 | 0 | 1 | 0 | 1 | 0 |
| 13 to <24 | 320/40 | 1 | 0 | 1 | 0 | 1 | 0 |
| 24 to <36 | 320/40 | 2 | 0 | 2 | 0 | 2 | 0 |
| 36 to <75 | 320/40 | 3 | 0 | 3 | 0 | 3 | 0 |

PPQ = piperaquine, DHA = dihydroartemisinin

**Block Randomization**

Identical slips of paper specifying treatment arm AL or DP will be put into an envelope. Each envelope will contain 20 slips from each group and children will randomly select one slip.

**Concomitant treatment and medication that should not be used**

Paracetamol will be used to treat fever. Iron and vitamin supplementation will be given if required. Prior treatment with an antimalarial drug will not be considered as exclusion criteria. Children taking any drug with antimalarial activity (including antibiotics such as tetracycline or azithromycin) during the study period will be withdrawn.

**Rescue treatment**

If children vomit twice, they will receive therapy with quinine intramuscularly and will be withdrawn from the study. Any child with signs of severe or complicated malaria will be treated according to recommendations of the attending nurse. If a child meets criteria for early treatment failure, he or she will receive parenteral quinine. If a child meets criteria for late treatment failure he or she will be re-treated with AL.

**4. Evaluation Criteria**

**Efficacy and safety evaluation**

Treatment outcomes will be early treatment failure, late clinical failure, late parasitological failure or adequate clinical and parasitological response as defined by the WHO[^2^](#_ENREF_2).

**Safety end points**

The incidence of any adverse event including neutropenia will be documented. All children will be asked routinely about previous symptoms and about symptoms that have emerged since the previous follow up visit. When clinically indicated, children will be evaluated and treated appropriately. All adverse events will be recorded in the case record forms.

Inclusion to the study will end if more than 40% of children in either study arm have reparasitaemias by day 14. This will be assessed in an interim analyses when 150 children have been included. If neutropenia (<1000 neutrofils per µl) is detected in more than 15 (10%) children the study will be stopped.

**Clinical evaluation**

All children will be evaluated by an attending study nurse and the examination will include a physical examination, body weight, body (axillary) temperature as shown in table 3

**Microscopic examination**

Two thick and thin blood films for parasite counts and species identification will be obtained as shown in table 3 and whenever a child returns to the Bandim health centre. Slides will be stained using freshly prepared Giemsa and examined with 1000 x magnification. The number of asexual parasites per 200 white blood cells will be counted or, if parasitaemia is high, the number of white blood cells per 500 parasites will be counted. Parasite density will be assessed independently by 2 qualified microscopists. Discordant results will be read a third time.

**Genotyping, antimalarial drug concentration, haemoglobin and white blood cell count**

100µL of blood will be collected on Whatman 3MM filter-paper using a capillary tube on day 0, 7, 14, 21, 28, 35, 42 and whenever reparasitaemia is detected. Filter-papers will be dried and then placed inside separate sealed plastic bags. Filter-papers and bags will be labelled with study number date and follow up day. The samples will be stored at -20˚C. Blood samples will be obtained at the same time that blood is taken for microscopy.

In order to differentiate recrudescence from a re-infection genotyping using sequential analysis of *pfglurp*, *pfmsp1* and *pfmsp2* will be done[^15^](#_ENREF_15).

The proportion of resistance associated genotypes in resistance associated genes including *pfcrt, pfmdr1, pfmrp1* and *pfcmu* will be determined. Genotyping will be done at Karolinska Institutet, Stockholm, Sweden and/or Universidade do Minho, Portugal.

Drug concentrations will be assessed on the week prior to reparasitaemia at Mahidol University Bangkok using high performance liquid chromatography (HPLC).

Haemoglobin concentration will be determined on day 0, 3 and 42 using a haemocue^TM^

At the same time that blood sampling for microscopy is done on Day 0, exactly 200μL of blood will also be taken for in vitro culturing from 50 patients. The blood will be put into medium and grown in vitro in an incubator at 37 ˚C. When a culture is established it will be frozen in liquid nitrogen for transport to Sweden or Portugal where extensive drug assays will be carried out.

**5. Study Assessment**

**Screening and enrolment**

As part of the routine medical services provided at the health centre, all children with symptoms suggestive of malaria will be screened for malaria using a rapid diagnostic test. Positive tests are confirmed by microscopy. Children fulfilling inclusion criteria will be invited to participate in the study following informed consent.

**Follow up**

Children will be given a unique personal identification number and be given treatment after informed consent. The basic follow up schedule is shown below. Treatments, information to the children, clinical observations and adverse events will be recorded in the clinical record forms. The children will come to the health centre on days 0, 1, 2, 3, 7, 14, 21, 28, 35 and 42. If a child is not seen at the health centre he/she will be visited at home.

Table 3. Study outline

| Action | Study Day | | | | | | | | | |
| --- | --- | --- | --- | --- | --- | --- | --- | --- | --- | --- |
|  | 0 | 1 | 2 | 3 | 7 | 14 | 21 | 28 | 35 | 42 |
| Clinical Records Form 1 | + | + | + | + |  |  |  |  |  |  |
| Clinical Records Form 2 |  |  |  |  | + | + | + | + | + | + |
| Standard physical examination | + | + | + | + |  |  |  |  |  |  |
| Body weight | + |  |  |  |  |  |  |  |  |  |
| Thick and thin smear$ | + | + | + | + | + | + | + | + | + | + |
| Temperature | + | + | + | + | + | + | + | + | + | + |
| 100μL of blood on filter-paper for genotyping or drug analysis* | + |  |  |  | + | + | + | + | + | + |
| Haemoglobin level (Haemocue™) | + |  |  | + |  |  |  |  |  | + |
| Differential count (Haemocue™ WBC DIFF) | + |  |  | + | + | + | + |  |  |  |
| 200μL of blood for in vitro culturing# | + |  |  |  |  |  |  |  |  |  |

$ Twice daily on days 0, 1 and 2. *The volume of blood spotted on to filter-papers should be exactly 100μL. # from 50 children

**6. Data Management**

The principle investigator will ensure that the study protocol is strictly adhered to and that data are correctly collected and recorded on the case record form. Data will be entered into an EpiData database during the study.

**7. Sample size**

The PCR corrected day 42 ACPR of AL was 97% in 2008 ( = before the national recommendation was changed in favour of A). It is likely that DP should be as efficacious as AL and it is therefore appropriate to conduct a non-inferiority trial[^13^](#_ENREF_13). Assuming 97% ACPR for both treatments, 5% significance level (alpha), 80% power (1-beta) and a non-inferiority limit (d) of 5% 144 children are required in each arm. Assuming a 20% loss to follow up a total of 346 children should be included.

**Analysis of data**

The data will be analysed using survival estimates of per protocol treatment failure rates but also intention to treat treatment failure rates. Final analysis will include a description of included children, proportions of adverse events and any serious adverse events, the proportion of children withdrawn or lost to follow up, the cumulative PCR corrected and uncorrected success and failure rates on day 42 and the proportion of early, late clinical and late parasitological treatment failures.

**Dissemination of results**

Results will be presented to the staff at the Bandim health centre and the ministry of Health and will be published in an international peer reviewed journal.

**8. Ethical considerations**

**Approval by the national ethical committee.**

The study has been approved by the ethical review board in Bissau, Guinea-Bissau (0021/CNES/INASA/2012). Molecular analyses in Sweden have been approved by the Stockholm regional review board (2011/832-32/2).

**Informed consent**

Children will only be included in the study if a parent or guardian gives informed consent. As the illiteracy rate is high, the consent request will be told by the study nurse using the local language Creole. Details about the trial including risks and benefits will be explained and any questions answered. The Clinical record forms will then be signed - if possible by the parent/guardian otherwise by the study nurse

**Confidentiality**

All information on the children will remain confidential and only shared by the study team.

**Health care services**

Recruitment is done at the Bandim health centre. All children not recruited will be treated according to standard practice. Study children will receive free health care throughout the follow up for any illness related to malaria regardless of treatment outcome. If children are found to have disease not related to malaria children will be referred for appropriate medical care.

**Inducement**

Health care and medications will be free during the study period but no other gifts or payments will be made.

**B. Effectiveness and safety of artemether + lumefantrine and dihydroartemisinin + piperaquine for the treatment of uncomplicated malaria in Guinea-Bissau**

**Objectives**

The primary objective is to measure the effectiveness and safety of AL and DP in children aged 6 months to 12 years suffering from uncomplicated *P. falciparum* malaria. Other objectives are as for the efficacy study except that haemoglobin will not be assessed on day 3 and neutropenia will not be assessed.

**Study design**

This study will be identical to the efficacy study with only minor differences as outlined below. Ethical considerations are the same in both studies.

**Timing and duration of the study**

The study will start when the efficacy study ends which will most probably be during spring 2014. The study will continue until a sufficient number of children are included. This is expected to take approximately 18 months.

**Treatment**

Randomisation and treatment allocation will be as for the efficacy study. Medication will be provided but there will not be observed intake and there will be no visit to the health centre on the afternoon of day 0 or on days 1, 2 or 3. Follow up, sampling and data collection will be identical from day 7 and onwards.

**Sample size**

The effectiveness of AL in our ongoing study appears to be approximately 80%. We expect the effectiveness of DP to be higher probably about 90%. Assuming a power (1-beta) of (80%) and a significance level of 5% we need 197 children in each study arm. Assuming a 20% loss to follow up a total of 474 children need to be included.

**References**

1. Wongsrichanalai, C., Pickard, A.L., Wernsdorfer, W.H. & Meshnick, S.R. Epidemiology of drug-resistant malaria. *Lancet Infect Dis* **2**, 209-218 (2002).

2. WHO. Guidelines for the treatment of Malaria. (2010).

3. Dondorp, A.M.*, et al.* Artemisinin resistance in Plasmodium falciparum malaria. *N Engl J Med* **361**, 455-467 (2009).

4. Noedl, H.*, et al.* Evidence of artemisinin-resistant malaria in western Cambodia. *N Engl J Med* **359**, 2619-2620 (2008).

5. Sisowath, C.*, et al.* In vivo selection of Plasmodium falciparum parasites carrying the chloroquine-susceptible pfcrt K76 allele after treatment with artemether-lumefantrine in Africa. *J Infect Dis* **199**, 750-757 (2009).

6. Sisowath, C.*, et al.* In vivo selection of Plasmodium falciparum pfmdr1 86N coding alleles by artemether-lumefantrine (Coartem). *J Infect Dis* **191**, 1014-1017 (2005).

7. Mwai, L.*, et al.* In vitro activities of piperaquine, lumefantrine, and dihydroartemisinin in Kenyan Plasmodium falciparum isolates and polymorphisms in pfcrt and pfmdr1. *Antimicrob Agents Chemother* **53**, 5069-5073 (2009).

8. Kofoed, P.E.*, et al.* Different doses of amodiaquine and chloroquine for treatment of uncomplicated malaria in children in Guinea-Bissau: implications for future treatment recommendations. *Trans R Soc Trop Med Hyg* **101**, 231-238 (2007).

9. Ursing, J., Kofoed, P.E., Rodrigues, A., Bergqvist, Y. & Rombo, L. Chloroquine is grossly overdosed and overused but well tolerated in Guinea-bissau. *Antimicrob Agents Chemother* **53**, 180-185 (2009).

10. Ursing, J.*, et al.* Similar efficacy and tolerability of double-dose chloroquine and artemether-lumefantrine for treatment of Plasmodium falciparum infection in Guinea-Bissau: a randomized trial. *J Infect Dis* **203**, 109-116 (2011).

11. Kofoed, P.E.*, et al.* Comparison of 3, 5 and 7 days' treatment with Quinimax for falciparum malaria in Guinea-Bissau. *Trans R Soc Trop Med Hyg* **91**, 462-464 (1997).

12. Davis, T.M., Hung, T.Y., Sim, I.K., Karunajeewa, H.A. & Ilett, K.F. Piperaquine: a resurgent antimalarial drug. *Drugs* **65**, 75-87 (2005).

13. 4ABC, T.f.a.-b.c.s.g. A Head-to-Head Comparison of Four Artemisinin-Based Combinations for Treating Uncomplicated Malaria in African Children: A Randomized Trial. *PLoS Med* **8**, e1001119 (2011).

14. Bassat, Q.*, et al.* Dihydroartemisinin-piperaquine and artemether-lumefantrine for treating uncomplicated malaria in African children: a randomised, non-inferiority trial. *PLoS One* **4**, e7871 (2009).

15. WHO. Methods and techniques for clinical trials on antimalarial drug efficacy: genotyping to identify parasite populations. (2007).
